# Trauma freed of the concept of determinism: is it possible to have a dialogue between psychoanalysis and neuroscience around the question of singularity?

**DOI:** 10.3389/fpsyg.2025.1529698

**Published:** 2025-05-09

**Authors:** Jessica Tran The, Emeric Saguin, François Ansermet

**Affiliations:** ^1^INSERM 1077, Neuropsychology and Neuroimaging of Human Memory, University of Caen, Caen, France; ^2^Agalma Foundation, Geneva, Switzerland; ^3^Department of Psychiatry, Bégin Military Teaching Hospital, Saint-Mandé, France; ^4^URP 7330 VIFASOM, Hôtel-Dieu, APHP, Paris, France; ^5^Department of Psychiatry, Faculty of Medicine, University of Geneva, Geneva, Switzerland

**Keywords:** neuroscience, trauma, Freud, post-traumatic stress disorder, interdisciplinarity

## Abstract

This article introduces a model of dialogue between psychoanalysis and neuroscience that is based on an account of the economic dimension of trauma. From the outset Freudian theory took into account the singularity of each subject’s response to traumatic events, setting aside any linear paradigm in the causality of symptoms. In 1980, the introduction of the nosographic category of PTSD (post-traumatic stress disorder) within the DSM (Diagnostic and Statistical Manual) contributed to an increased social recognition for sufferers. Yet, it also resulted in a form of standardization in a clinical picture that hitherto had been heterogenous. The result was a deterministic and linear epistemological paradigm whose effects could be normative. Once we have defined the opposition between these two paradigms, we propose demonstrating that a dialogue is possible between psychoanalysis and neuroscience around the concept of ‘trauma’. To do this we will introduce an interdisciplinary approach that is free of the pitfall of determinism, and that seeks to promote the consideration of singularity in clinic practice. From that perspective, the post-traumatic symptom is no longer viewed as the consequence of a particular event, rather it is a construct produced by the subject in their effort to manage what overwhelms them.

## 1 Introduction

Since its beginnings, psychoanalysis has maintained a complex relationship with the concept of psychological trauma. It is a concept on which Freudian theory evolved in a twofold movement of affinity followed by antonymy. Although Freud’s experiences with his first hysterical patients in the 1890s had initially given rise to a theory of the traumatic etiology of hysteria—where the symptom was attributed to a real ‘scene of sexual seduction’ (Freud, 1896)—the birth of psychoanalysis is commonly fixed on September 21, 1897, when Freud confided in Fliess the ‘great secret’ that had slowly revealed itself to him, ‘I no longer believe in my neurotica’ (Freud, 1898, p. 264).

The outcome of abandoning his theory of a traumatic etiology in hysteria, was the proclamation of a relationship of strict equality between the etiological values of fantasy and any real traumatic scene experienced in childhood (Freud, 1898). This change of heart should not be seen as bringing into question the truth of neurotic speech, nor the existence of real sexual abuse (which Freud did not deny) (Freud, 1900); rather, it is a shift in the pathogenic process. Freud was turning away from exogenous factors to consider the endogenous trigger in the formation of symptoms. He noticed that some seduction scenes reported by his patients were based on screen memories—whose function is to form a defense against the memory of the patient’s own infantile sexual activity, and the oedipal fantasies that accompany it (Freud, 1900). This recognition of the psychic reality and the pathogenic power of fantasy in the formation of symptoms, implies that the impact of external events on the subject’s life can no longer be studied without taking into account the endogenous action of drives and the singular psychic positioning of each person in the face of lived experiences.

Freud’s discovery also inflected the paradigm of sexual trauma in a direction where sexuality was, of itself, universally traumatic—being in effect a form of otherness within the psychic apparatus itself, wherein ‘the majority of the *driving*^1^ demands of this infantile sexuality are treated by the ego as dangers’ (Freud, 1926, p. 155).

Among post-Freudians, the French psychoanalyst Jacques Lacan stressed that behind this first impression analytical experience quickly revealed that the introduction of sexuality—through the encounter with the unassimilable reality of the sexual drive—was a universally experienced trauma. Furthermore, for the subject, this trauma had ‘an organizing function for development’ (Lacan, 1981, pp. 55, 64). Through the endogenous excitation of the drive, both the otherness and the interiority of trauma come together. Oedipal desire constitutes a first attempt to symbolize this excitation by way of representations connected to the other, but it also irredeemably alienates the desire of the subject from the desire of the other. Lacan insisted on the traumatic aspect inherent in this relationship to desire. The desire of the other remained as a potentially traumatic ‘enigmatic nucleus’ until, in the aftermath, the subject could reintegrate it into a signifying chain—a chain of meaning that then constitutes the ‘nucleus’ of neurosis (Lacan, 2019, p. 500).

Given that turning away from an external traumatic etiology for neurotic disorders played a part in the birth of psychoanalysis, it comes as no surprise that Freud foresaw how, in the aftermath of the First World War, debates around traumatic neuroses in soldiers would bring grist to the mill of ‘the opponents of psycho-analysis, whose repugnance to sexuality has shown itself to be stronger than their logic’ (Freud, 1920, p. 2).

In this article we will explore how, since the 1960s, the increase in research on the psychic consequences of confronting certain events considered traumatic has indeed led to the advent of a paradigm that goes against the psychoanalytic perspective. We observe that, counter to the individuality and the great heterogeneity of clinical manifestations observed following a confrontation with a traumatic event, the introduction in 1980 of the nosographic category of PTSD (post-traumatic stress disorder) within the DSM (Diagnostic and Statistical Manual) not only operated a form of standardization of clinical pictures, but was also underpinned by a deterministic and linear epistemological paradigm. As we shall demonstrate, Freudian theory had from the outset looked beyond a linear paradigm in the causality of symptoms, to favor the individuality of the subject’s response to traumatic events. Once the opposition between these two positions has been explored, we propose laying the foundations for a dialogue between psychoanalysis and neuroscience around the concept of trauma. Specifically, we propose introducing a model of dialogue that revisits the fundamental economic dimension of trauma. Seen in that light, the post-traumatic symptom is no longer viewed as the consequence of a particular event, rather it is a construct produced by the subject in their effort to cope with what overwhelms them. In that context, and despite their heterogeneous epistemological foundations, psychoanalysis and neuroscience may find common ground around an understanding of the singularity of the subject’s response regardless of what determines them.

## 2 Creation of the nosographic category of PTSD: a social genesis, rather than a medical one?

Since the late 1960s, Western societies have experienced a reversal of paradigm as regards the concept of trauma. Fassin and Rechtman have traced the genealogy of this shift, identifying when trauma took on its significant position in the moral sphere of contemporary societies. If suspicion once weighed on military or civilian victims of traumatic events, whose claims were most often considered illegitimate—for instance soldiers decried as cowards or deserters—recognition of trauma has now reached all strata of our societies (Rechtman and Fassin, 2011). The identification and recognition of this status now forms the basis of contemporary social welfare systems’ treatment of trauma—including access to benefits, and financial compensation rights.

The emergence of a less negative social discourse surrounding psychopathological symptoms consequent to exposure to traumatic events came about in 1980, with the introduction of the new nosographic category of PTSD, within the DSM. This new perception of trauma was not driven by an evolution in legal and military psychiatry, but by changes that were played out on the moral and socio-political stage. This emergence was strongly influenced by social rights movements—Vietnam veterans, women victims of sexual violence. In that regard, it resulted from the mobilization of actors outside the field of mental health (Rechtman and Fassin, 2011).

In the 1960s the issue of child abuse became a political priority in the U.S. at the same time fueling criticism against Freudian theory. The main objective of the 1971 conference given in New York on April 17 by the feminist social worker Florence Rush, was to lift the veil on sexual abuse of children; argumenting that child abuse, often sexual, prefigures the fate of women in society. She denounced a ‘conspiracy of silence’ among public authorities, including psychiatrists (Rush, 1980). The experience of survivors of incest was compared to that of Holocaust survivors: the same wholesale denial, and the impossibility to put into words the horror experienced. Whereas previously trauma belonged to an individual and subjective experience, it now became a universal representation of human vulnerability. The discovery of the horrors of the camps had brought about a collective awareness of the devastating consequences of psychological trauma (Rechtman, 2002).

The increase in visibility of the movement for the recognition of victims of sexual and child abuse, was accompanied by a full-on criticism of the Freudian theory of fantasy. Rush argued that if Freud had been right with his first etiological theory of hysteria, his subsequent abandoning of his neurotica was due to a lack of courage in the face of the real magnitude of sexual abuses (Rush, 1980). This thesis found echoes within the psychoanalytic movement itself (Fliess, 1973; Masson, 1998).

The first and second editions of the DSM reflected in their nosography the influence of the psychoanalytic approach to psychopathological disorders—notably through the bipartition between neuroses and psychoses. After 1973 however, the American Psychiatric Association (APA) undertook to revisit its classification, entrusting the project of a new edition of the DSM to Robert Spitzer. The challenge was to modify both the headings for the nosographic categories, and the main etiological hypotheses—with the intention of removing scientifically unproven concepts. The motivations for this reform were marked by important social and moral issues. The DSM-III wanted to demonstrate the capacity of psychiatry to defend those parts of the population oppressed by the social order, when previously it had been accused of legitimizing or reenforcing that oppression (Rechtman and Fassin, 2011). Most notably, Spitzer obtained the removal of the diagnosis of homosexuality (Minard, 2013). One of the main ethical challenges was a desire to redefine mental illness independently of any moral judgment—psychiatric diagnosis having previously contributed to a form of social control. The DSM-III was published in 1980, for the first time in the history of psychiatry the new names, hypotheses, and ideology conveyed resonated with the needs and expectations of its users. This was evidenced by the new place given to psychological trauma, and the new nosographic category of PTSD (American Psychiatric Association, 1980a,b).

The semiology of PTSD picked up some aspects and symptoms linked to ‘war neurosis’ while ending the suspicion that surrounding it. In particular, the DSM-III foregrounded ‘Criterion A’: Exposure to actual or threatened death, serious injury, or sexual violence’ be it directly or indirectly. Positioning the traumatic event as the first diagnostic criterion opened the way to the social recognition of victims and to financial compensation, for instance for veterans who until then had not obtained any recognition (Rechtman and Fassin, 2011). The impact was also observed elsewhere.

**Figure 1:**
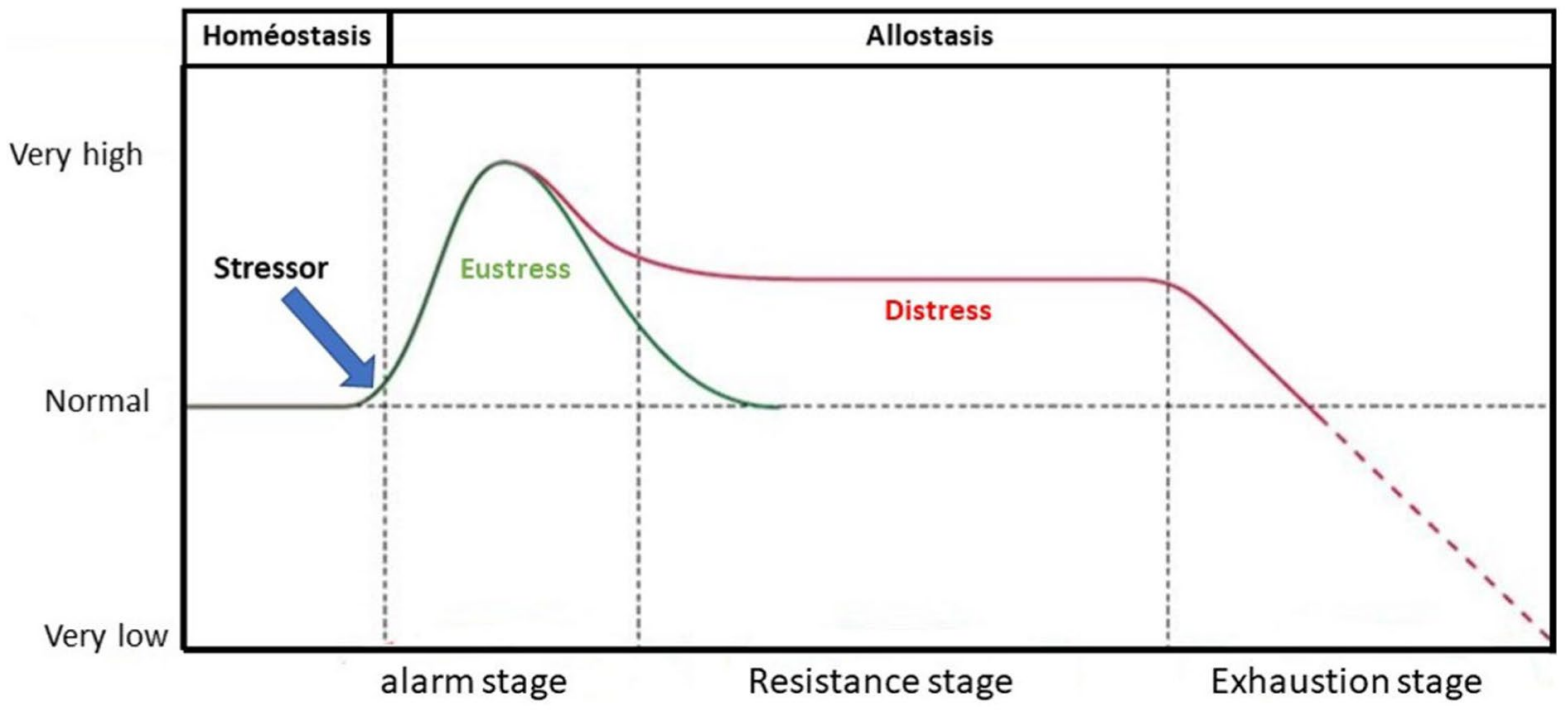
Activation level of different reactions characteristic of stress (adapted from Selye, 1950).

This new nosographic category, while responding to social demand, drew its scientific legitimacy from the Selye’s stress model, derived from physiology, and involving three distinct phases that follow each other in a linear and universal manner: a first phase of neuroendocrine alarm involving catecholaminergic and then corticotropic responses; a second phase of adjustment of the stress response to the strictest need; a third phase that sees depletion of biological resources leading to death or the emergence of pathologies (Selye, 1950). Lazarus and Folkman formalized an application of Selye’s model by proposing to redefine stress as the result of a dynamic interaction between an individual and their biological and psychological resources, in the face of environmental demands, by way of various coping strategies (Folkman and Lazarus, 1985). During stress, the body faces constraint at the cost of expensive biological mobilization (allostasis). This cost corresponds to a production of energy and the degradation of metabolic waste (catabolism), actions that are carried out at the expense of the reconstruction of the body’s structure (anabolism). This metabolic impact is the consequence of impregnation with stress hormones, glucocorticoids. These facilitate glucose production at the expense of protein synthesis and catecholamines, promoting lipolysis and glycogenolysis (Figure 1).

The concept of allostasis, essential to this model, differs from classical homeostasis: in contrast to the Bernardian idea, allostasis presupposes a close dependence of the organism on the environment, thus emphasizing the need for adaptation (Arminjon, 2014). Where Bernardian constancy included the existence of internal stimulants that the body had to strive to regulate (Bernard, 2013), contemporary concepts of regulation lead to a focus on the body’s relationships to environmental stimulants. PTSD is thus underpinned by a scientific model describing the body’s extreme response to intense stress, which would lead to a lasting dysregulation of the stress control system.

## 3 PTSD: the standardization of a clinical heterogeneity

While the social consequences of introducing PTSD are undeniable, there was also a clinical effect: the standardization in the heterogeneity of psychopathological manifestations. In effect, this new diagnosis subsumed a plurality and diversity of clinical realities within a single diagnostic entity. In 1980 Summerfield noted a reduction in the heterogeneity of diagnoses posed by American psychiatrists on veterans. Prior to that, diagnoses were extremely varied (anxiety, depression, consumption or abuse of toxic substances, schizophrenia in some cases), thereafter they would all be subsumed within PTSD (Summerfield, 2001).

It should be emphasized that, historically, the semiology associated with post-traumatic psychopathological states had presented itself in an extremely polymorphic manner. As early as antiquity, there were accounts of functional damage to certain organs without any anatomical lesions being present. One of the earliest cases,: at the Battle of Marathon the Athenian Epizelus ‘while he fought doughtily in the melee lost the sight of his eyes, albeit neither stabbed in any part nor shot’ (Herodotus, 1922, p. 271). Another case is that of the ‘distinguished soldier’ in whom Pinel diagnosed an attack of hypochondria: clinically, he presented with ‘spasms in the limbs, waking with starts, frightening dreams, and sometimes erratic heat in the feet and hands […] if he hears of any disease […] immediately believes he is a prey to it’ (Pinel, 1809, p. 32) [our translation].

From 1880, although the nosography of post-traumatic psychopathological states crystalized around the definition of new diagnostic entities. In the clinical picture of ‘fright neurosis’ (Schreckneurose), Kraepelin described the predominance of anxious, depressive, and psychosomatic symptoms, and in some cases hallucinations and delusions (Kraepelin, 1889). Oppenheim introduced the term ‘traumatic neurosis’, outlined forty-two cases of psychopathological states following work accidents and described a wide variety of symptoms: generalized anxiety, motor disorders, including tremors, somatic pain, and paralysis of certain limbs without spinal cord injury (Oppenheim, 2012).

Beginning with the First World War, we witness a number of competing semiological and nosographic descriptions of post-traumatic clinical pictures in soldiers: chronic convulsive tremors; “battle hypnosis”; rhythmic myoclonus; vomiting; camptocormia (bent spine); enlarged stomach, “meteorism,” “tympanism”… (Roussy and Boisseau, 1917; Loisel and Saguin, 2021). Coming from a different viewpoint, psychoanalysts contemporary to Freud who served as military psychiatrists in 1914–1918 were also highlighting the polymorphic nature of the clinical pictures they observed in soldiers. Ferenczi argued that a distinction should be made among war neurotics, between patients who suffered from various ‘monosymptomatic’ conditions (including pain or paralysis of a limb unrelated to organic damage), and those—much more numerous according to him—who had generalized tremor and gait disorders (Ferenczi, 1916). In contrast with this heterogeneity, today’s PTSD focuses on four semiological criteria, these are: intrusion symptoms, e.g., recurrent dreams (Criterion B), avoidance of stimuli associated with the traumatic event (Criterion C); mood disorders, e.g., reduced interest in certain activities (Criterion D); as well as profound changes in wakefulness and responsiveness, e.g., tantrums, hypervigilance, startle response, or sleep disturbances (Criterion E). We may note that Criteria D and E are common to other diagnoses, such as mood disorders and sleep disorders (American Psychiatric Association, 2013).

Although originally strongly influenced by a humanistic vision of psychiatry, which claimed to no longer be a tool of social control, the introduction of the diagnosis of PTSD has, in the long term, prompted normative effects. The symptoms of criteria B, C, D, and E have become to some extent the ‘normal’ consequence of a healthy person’s exposure to an ‘abnormal’ event (Rechtman and Fassin, 2011). This perspective may be a result of Krell’s focus on Holocaust survivors: in his words, ‘to be sane after the camps is not sane’ (Krell, 1984).

By revealing the psychological consequences of exposure to particularly violent events, the introduction of this new syndrome formed part of a desire to denounce the universal impact of tragic and violent circumstances such as wars. PTSD, introduced in a textbook that claims to be statistical, implies reference to a normal law in the mathematical sense: the distribution of symptoms listed under criteria B, C, D, and E would be the most frequent from the point of view of their probability density. Nevertheless, this statistical standard also has a normative effect. As Canguilhem (1966) had observed, ‘normal’ implies ‘exhibiting’ that norm, reproducing the rule as well as pointing to it. Yet a norm cannot be thought of without accounting for what it ‘leaves out’, ‘A norm draws its meaning, function and value from the fact of the existence, outside itself, of what does not meet the requirement it serves’(p. 239). The PTSD, by indicating a ‘normal’ model of the consequences of a violent event, acts as both normative and normalizing. The statistically ‘normal’ reaction to the event described by the nosographic category PTSD ultimately induces prescriptive effects, and erase the reality of clinical diversity. Hacking (2013), focusing on the epistemological and social consequences of the DSM, extended this prescriptive aspect by stating that standardization tends to mold a typical clinical picture. With its cocktail, or ‘menu’, of symptoms, a diagnosis can influence or even prescribe how patients perceive themselves: ‘especially since nowadays, when told their diagnosis, patients tend to look it up online. There they get a sort of stereotype of how they ought to be feeling and behaving’ (Hacking, 2013). Clearly, the introduction of PTSD marked a definitive break from the previous model of traumatic neuroses; and the decision to eliminate the term ‘neurosis’ from this diagnosis sealed the abolition of more than a century of suspicion directed at victims. Yet this new categorization acts like a pharmakon, it is both a remedy and a poison (Derrida, 1989). It induces a form of universalization that tends to subsume or even erase singularity. For the subject, it can then become a trap, similar to that which Freud had described in The Future of an Illusion where he wrote, ‘Their acceptance of the universal neurosis spares them the task of constructing a personal one’ (Freud, 1927, p. 44).

## 4 The etiological power of the event, cornerstone of a deterministic and linear epistemological paradigm

To find that a nosographic category subsumes heterogeneity within an artificial classification is certainly not unique to the diagnosis of PTSD. It is one of the defining aspects of classificatory medicine and is characterized by what Foucault identified in *Birth of the Clinic* as an analogical principle. Doctors proceed by analogy, grouping similar symptoms within one category of disease. Thus, “kinship folds back into identity” (Foucault, 1975, p. 7). A category is thus built in the negation of the uniqueness of each pathology. The patient and their singularities constitute an obstacle for the clinician in accessing the “pure nosological essence”, since ‘the patient adds, in the form of so many disturbances, his predispositions, his age, his way of life, and a whole series of events that, in relation to the essential nucleus, appear as accidents’. In such a way that to arrive at the truth of the pathological fact, “the doctor must abstract the patient” (Foucault, 1975, p. 8).

Yet, whereas an analogical method usually involves first collecting symptoms that seem similar and then looking for a common cause, what seems specific to the standardization induced by the nosographic category of PTSD is the fact that it is the hegemony of a common cause—the confrontation with a traumatic event—that presides over the grouping and homogenizing of disparate symptoms. In this regard the diagnosis of PTSD stands out within the DSM-III: it is the only nosographic category that is not only descriptive but also etiological—with the mention in first position of Criterion A, encompassing the different types of exposure to traumatic events. Usually, the etiology—otherwise considered as multifactorial—is not included in the DSM definitions (Rechtman and Fassin, 2011).

Criterion A is the perfect guarantor of the new moral imperative to acknowledge victims. This is evidenced by the fact that attacking the exclusive etiological power of Criterion A—as Summerfield (2001) did by claiming that earlier factors could play a major role in explaining the wide variability of symptoms—immediately unleashes the outrage of victim associations. Indeed, Criterion A, as a necessary or even sufficient condition for the occurrence of symptoms, becomes the *inventio medii*,^2^ making it no longer possible to consider the psychopathological picture as the consequence of a fragile, cowardly, or complaintive personality. On the contrary it is a ‘normal’ reaction—in the statistical sense—to the event.

Once the traumatic event is given as an etiological agent, this nosographic category becomes rooted in a deterministic epistemological correlate: the symptoms described by Criteria B, C, D, and E appear as direct *consequences* of exposure to the event (Criterion A), which thus acquires the status of *cause*. This nosographic category, which in 1980 claimed to be at the forefront of both scientific advances and contemporary social issues, was therefore paradoxically underpinned by a conceptual framework that dated from the eighteenth century. The idea of determinism had appeared with classical physics in the context of the study of cause-and-effect relationships, to describe a succession between a first and a second event, as governed by a relationship of necessity. The physical idea of determinism covers not only a causal relationship according to which the cause produces the effect, but also a ‘legality’ relationship according to which by knowing the cause one also knows the effect (Kojève, 1990, pp. 46–47). This legality component of physical determinism is linked with the ideal of prediction; something that Laplace, through his famous formula,^3^ focused on as the state to which science should aspire.

However, the advent of modern physics, and the discovery of quantum mechanics in the 1930s, rendered obsolete the absolute determinism of dynamic systems, and the possibility of their predictability (Prigogine and Stengers, 1984). In particular, the principle of indeterminacy (*Unbestimmtheit*) proposed by Heisenberg describes the absence of an unambiguous numerical determinant of a physical quantity. This implies the physical impossibility of accurate and complete individual forecasts. However, the nosographic entity of PTSD, listing different categories of symptoms that are the effect of a single cause pinned down by Criterion A, implies a return to a causal and deterministic epistemological paradigm. This linear model discards the possibility of a discontinuity occurring between the event and the symptom.

The physiological model of stress that would give the diagnosis of PTSD its scientific basis, also implied a deterministic and linear perspective. Initially, this model referred to both a clinical and biological understanding, and did not involve a pathological dimension. The stress response was useful in coping with a threat, but it would disappear with the cessation of exposure to the stressor. However, Selye’s experiments had demonstrated the absence of a quantitative correlation between the triggering constraint and the biological response (Selye, 1950). The concept of stress subsequently underwent a gradual semantic shift, causing confusion between cause and effect. ‘Stress’, which initially described a physiological mechanism of constraint that was part of an adaptive response, came to mean a dysregulation of the organism. This physiological dysregulation would then be understood as the initial pathological mechanism of PTSD. This reversal of the paradigm meant that stress was no longer understood as a consequence of the stressor’s constraint, but as a causal biological determinant of the disease. The reaction had now become the cause. And where in Seyle’s model the focus was on the organism, it now shifted onto the traumatic event. This shift reinforced the role of stress as the main etiological factor in the symptomatic picture, and made psychological trauma a purely ‘reactive’ pathology.

The stress model’s main limitation continues to be its continuist nature. By focusing on a linear response to the causal factor of stressors, this model overlooks the sudden nature of, and the discontinuity, produced by a traumatic event (McFarlane, 2010). Moreover, through excessive simplification, this model struggles to account for the extreme interindividual variability of reactions after a traumatic event, and intraindividual variations over time (Bonanno, 2008). Despite efforts to integrate an increasing number of biological and psychological dimensions into the stress model, by removing the dimension of psychic causation the model continues to underestimate the complexity of the dynamic interaction between these two fields (Rutter, 2012). The idea of the cumulative effect of exposures, and the observation that an individual’s reactions are amplified by a history stress, have now been integrated into the model (Binder and Holsboer, 2012). Yet, in this logic of the double hit, the initial exposure to a stressor is perceived as an acquired vulnerability. This is a vulnerability that, when followed by the second exposure to another stressor, becomes responsible for the appearance of the symptomatology. While this model of vulnerability highlights the importance of the subject’s history in the emergence of a psychopathological picture, the idea of biological vulnerability introduces an *a priori* biological determinism: the biological vulnerability reemerges at the time of re-exposure. In that respect, the stress model is dependent on a logic of vulnerability and predictiveness that remains trapped in the epistemological paradigm of classical physics.

We will now clarify how, from an epistemological point of view, the Freudian understanding of trauma goes against the linear model.

## 5 The Freudian thesis of overdetermination in mental pathologies: a critique of linear determinism

The historical social context in which the nosographic entity of PTSD emerged was accompanied by a strong criticism of Freudian theory; and once this new syndrome was established, it signaled an epistemological opposition to the psychoanalytic model. Very early the Freudian theory had criticized the hypothesis of a linear determinism in the etiology of mental illnesses. Beginning in 1896, Freud had opposed the idea of monofactorial causation in psychopathologies, insisting on the meshing between innate and acquired factors in the genesis of symptoms (Freud 1896). In 1917, he chose the term ‘complemental series’ (Freud, 1916-1917b, p. 362) to designate this combining of different levels of causation. An initial complementary series involving the interaction between exogenous and endogenous factors in determining of the subject’s libidinal fixations consisted of: innate dispositions of the sexual affects, infantile history, and the influence of fantasy life on libidinal development. This series that culminates in the formation of the subject’s ‘constitution’—(Struktur) (Freud, 1933, p. 59)—can subsequently be combined with the occurrence of an ‘accidental’ experience in adult life. That association would then form a second complementary etiological series in the genesis of the symptom (Freud, 1916-1917b).

Freud attributed the fundamental human tendency to gravitate toward single and deterministic causality for psychic pathologies to ‘the restricted nature of what men look for in the field of causation: in contrast to what ordinarily holds good in the real world, people prefer to be satisfied with a single causative factor’ (Freud, 1912, p. 99). We, in turn, might stress that the ‘single cause’ varies depending on historical and scientific contexts.

In opposition to a monocausal explanation—which could be said to fall into a *Weltanshauung*, a unifying, systematized and fixed worldview—Freud always argued for the ‘overdetermined’ character of the symptom (Freud and Breuer, 2004). For Freud, the symptom was the result of a plurality of causal chains: “We refuse to posit any contrast in principle between the two sets of aetiological factors; on the contrary, we assume that the two sets regularly act jointly in bringing about that observed result. ∆αί μων καі Tύχη^4^ […] determine a man’s fate—rarely or never one of these powers alone” (Freud, 1912, p. 99). Any prospect of attributing the cause of the subject’s symptoms to exposure to a traumatic event was therefore contrary to the Freudian epistemological model.

Freud explicitly criticized the dangers posed by a representation that viewed the symptom as a consequence of the introduction of an external pathogen or ‘foreign body’. He denounced a model that hinged on “some bacillus which could be isolated and bred in a pure culture and which, when injected into anyone, would invariably produce the same illness” (Freud, 1926, p. 153). On the same occasion Freud once again decried any aspiration to seek a final, tangible, and homogenous cause.

## 6 The deferred action model: a shift away from the traumatic event in favor of the discontinuity of the subject’s individual response

When we say that monofactorial causality was foreign to Freud’s thinking, we should emphasize that^5^ even when he argued that a real trauma—particularly a scene of sexual abuse occurring in childhood—would contribute to the formation of a hysterical symptom, he did not conceive this incidence as a linear cause-and-effect model. On the contrary, he introduced the concept of ‘deferred action’ to highlight the significant influence of temporality in the process. This is decisive in understanding the radical distance between psychoanalysis and other theories that sought to locate the pathogenic power of trauma within an event.

It was in A Project for a Scientific Psychology (Freud, 1985), while analyzing his patient Emma’s phobia of entering a shop alone, that Freud brought to light how trauma always presupposes the existence of at least two events. In an initial scene, called ‘seduction scene’, the child experiences sexual solicitation from an adult without this giving rise to sexual excitement. A second scene, often seemingly innocuous and occurring after puberty, then evokes by some associative trait that first scene (Freud, 1985, p. 354). Freud stated that this two-stage temporal process always occurs in the formation of hysterical symptoms where ‘we invariably find that a memory is repressed which has only become a trauma by deferred action (*Nachträglichkeit*)’ (Freud, 1985, p. 356).

This Freudian theory of deferred action introduced several major innovations. On the one hand, if it is the memory of the first scene that triggers an influx of excitement that swamps the ego, it does not act owing to its own energy, it only acquires its traumatic power in the aftermath of the second event. Freud attributed this delayed incidence very specifically to ‘the retardation of puberty as compared with the rest of the individual’s development’ (Freud, 1985, p. 356). Even at the time of his neurotica the germ of the idea of a universally traumatic essence, linked to sexuality, was present in Freud’s thinking. According to Freud the influx of sexual arousal—source of unpleasure which triggered the process of repression—was an arousal that originated internally. It was its discharge that constituted the traumatic element. Freud describe this ‘quantitative factor’ as having its source not in the external event—the seduction scene—but in an event of an endogenous nature, namely the early awakening of the drive^6^ in infant sexuality (Freud, 1985). Freud maintained this theory throughout his work, reformulating it in 1926 in the context of his final theory of anxiety where he states that ‘The majority of the *driving* demands of this infantile sexuality are treated by the ego as dangers’ (Freud, 1926, p. 155). The reason being that a child’s psychic apparatus would not yet be up to ‘mastering the quantities of excitation which require to be disposed of’ (Freud, 1926, p. 148).

The concept of deferred action highlights the importance of temporality in the formation of symptoms and abolishes any ‘summary interpretation which reduces the psycho-analytic view of the subject’s history to a linear determinism envisaging nothing but the action of the past upon the present’ (Pontalis and Laplanche, 1988, p. 112). Thus, the term ‘deferred action’ concealed a significant heuristic aspect. Since, as early as 1895, it consummated the opposition between Freudian trauma theory and any model that postulated a single linear determinism linking a traumatic event acting as ‘cause’, with a symptom that would be its direct ‘effect’. Thereupon, psychoanalysis could not be reduced to a theory that argued that the destiny of a human being was decided in the first months of their infancy (Pontalis and Laplanche, 1988). Freud had, it is true, argued for the existence of a psychic determinism in the inscription of experience in the form of a trace, with the idea that everything is retained in the psychic apparatus—like the city of Rome where the remains of the past perdure (Freud, 1930). Yet, in his letter to Fliess of December 6, 1896, he had also described a psychic mechanism of reassociation of traces, through which ‘the material present in the form of memory traces [was] subjected from time to time to a rearrangement in accordance with fresh circumstances’ (Freud, 1896, p. 207). The different memory traces, which had originally been associated with each other according to a criterion of simultaneity, could subsequently be reassociated within the psychic apparatus in accordance with the subject’s new perceptual experiences.

Bistoen et al. (2014) had enlighted that the Freudian concept of *Nachträglichkeit* is central to the psychoanalytical understanding of trauma (2014). This concept offers an interesting perspective on both the well-established yet controversial finding that traumatic reactions sometimes follow in the wake of non-Criterion A events (so-called minor stressors or life events), and the often-neglected phenomenon of delayed-onset PTSD. The delayed-onset PTSD diagnosis has two possible definitions: a considerable delay in the emergence of symptoms, or the presence of early symptoms that are not very specific and not attributed to the effects of the event on the psyche. Since different traumatic experiences share common elements, each new event with psychotraumatic potential would activate the same memory structure, which would reinforce the interconnections of this memory network. This could explain the role of a later triggering event in the development of delayed post-traumatic disorders (Annette et al., 2023). As Annette and her collaborators pointed out, according to the Freudian concept of *Nachträglichkeit*, the first event is initially without consequence, but it is transformed by a second event, and it becomes retroactively traumatic. Either the first event left an unsymbolized trace—it was not integrated into the signifying chain, and it was subsequently integrated later on due to the second event—or the first event was symbolized at the moment of its occurrence, and in the light of a second subsequent event, it assumed a new meaning (Annette et al., 2023). However, it is not necessarily the time of occurrence of the event that would play the most important role, but rather its content. Life events that recall the original traumatic event, in a real or symbolic way, could activate and highlight the latent psychopathology. It could be a situation of daily life, a word, a sensation, or events such as a romantic breakup, an experience of professional failure, and sometimes even a happy event that is apparently far removed from the circumstances of the initial trauma (Annette et al., 2023; Auxéméry, 2021). Delayed-onset PTSD highlights the shifting and indeterminate nature of psychic life, and the difficulty in predicting post-traumatic stress disorders. *Nachträglichkeit* clarifies one way in which traumatic encounters are mediated by subjective dimensions above and beyond the objective particularities of both the event and the person. It demonstrates that the subjective impact of an event is not given once and for all but is malleable by subsequent experiences (Bistoen et al., 2014).

The implication of the Freudian thesis for the reworking of traces was that once inscribed within the psychic apparatus the memory trace would not be fixed and immutable. Traces could be subject to a certain lability and be reassociated with new representations in the wake of new experiences. These subsequent reassociations would then contribute to distancing the new representations from the initial perceptual traces, introducing a discontinuity between the experience and its inscription. It is precisely within this discontinuity that the possibility of freedom for the subject can emerge. The subject would no longer be purely the passive product of the experience that was inscribed in them (Ansermet and Magistretti, 2010). Therefore, although the mechanisms of the psyche are influenced by our past experiences, a permanent openness to contingency introduces a space within which the freedom of the subject can find expression. This allows psychoanalysis to function as a therapeutic technique: not being condemned to remain the pure passive product of their infantile history the subject can instead have a degree of choice, giving them back an active role in the shaping of their future. Rather than the trace left by past experience in our psyche, psychoanalysis is interested in the subject’s position vis à vis traces that are severed from the initial perceptual experience following successive rearrangements.

## 7 R – S: a model for a dialogue between psychoanalysis and neuroscience

Psychoanalysis agues the existence of a discontinuity between event and symptom, whereas it is a continuist epistemological paradigm that underpins the diagnosis of PTSD in the DSM-III. Does this mean that a dialogue between psychoanalysis and contemporary scientific advances around the concept of trauma is impossible?

We argue that it is possible to establish the foundations of an interdisciplinary dialogue between these disciplines regarding the psychopathology of trauma, based on different neuroscientific works that contribute to go beyond a deterministic conception of memory. In particular, current research on the relationships between the hippocampus and the regions involved in emotions and the perception of body states, but also discoveries relating to the cellular and molecular mechanisms of memory reconsolidation. What we propose, is to approach the field of trauma from the perspective of an interdisciplinary model we have developed and called ‘R – S’ (Representation – Somatic State), and that we will describe. This paradigm may also have some similarities with Damasio’s somatic marker theory and Friston’s free-energy principle.

Researchers have suggested bringing together recent neuroscientific work on memory and the Freudian concept of deferred action. Humans typically cannot remember events occurring during the first 3 years of life. But this phenomenon has intrigued scientists: do people not remember experiences from infancy because the hippocampus is too immature to form memories, or are these memories formed and then forgotten? (Ramsaran and Frankland, 2025) Neurobiological data highlight that the hippocampus, as the seat of declarative memory, is not functional before the age of three. This leads us to conclude that early memories involve a reconstruction, one in which fantasy plays a primary role (Yovell, 2000). But recent study provides functional neuroimaging evidence that the infant hippocampus can encode the types of information that are required for episodic memory, that is, memories for specific life events containing information about people, places, and things. This suggests that immaturity of postencoding processes, such as memory consolidation or retrieval, is a more likely explanation for infantile amnesia (Yates et al., 2025). Based on the distinction between emotional amygdala-based memory and hippocampus-based declarative memory, Ledoux (2005) suggests that through the effects of stress, traumatic events have a neurotoxic effect on the hippocampus. What results is a strengthened inscription in the amygdala; and thus an implicit, non-conscious memory, which is then combined with an absence of explicit memory. Edelman’s (1989) theory of reentry circuits, or Damasio’s theory of zones of convergence and divergence (Meyer and Damasio, 2009), both argue that memory is always the result of a deferred cortical reshaping (Cabelguen and Rabeyron, 2019).

Since the 2000s, discoveries related to work on neural plasticity have led neurobiologists to conclusions that agree with Freudian ideas on the discontinuity introduced by the reassociation of traces (Ansermet and Magistretti, 2007; Tran The, 2020). Electrophysiological studies show that newly learned information is encoded in the brain as patterns of neuronal activity (Eichenbaum, 2004). With time, this information is transformed into more persistent modifications, which seem to be engrained in molecular or structural forms such as structural modifications of existing synapses or formation of new ones (synaptic plasticity). This process of transforming the activity induced by new learning into stable, long-lasting modifications has been termed memory consolidation (McGaugh, 2000). An important feature of the memory consolidation process is that for a limited time after learning, the new trace is labile because it can easily be disrupted by several types of interfering events. Experiments have shown that if a new memory is exposed to challenges such as brain trauma, seizure, a second learning event, or pharmacological treatments of many sorts, it fades away, and recall tests at later times show amnesia. This has been found in a multitude of types of memories and animal species including humans (Squire et al., 2004). With time, however, the memory becomes increasingly stable until it is fully insensitive to disruption or consolidated. Indeed, if the interfering challenge is presented some time after the memory is formed, no effect is seen, and the memory survives perfectly. Hence, there is an opportunity for disrupting newly formed memories immediately after they are formed and for a limited time. How long does this time window of opportunity last? The answer to this is still debated. General interfering events, such as traumas or brain lesions, suggest that memory consolidation takes a relatively long time, which although variable in different memories, can take several years in humans. On the other hand, pharmacological and molecular interferences, such as an acute blockade of *de novo* protein synthesis, disrupt memories only if applied soon after training, but they are ineffective a few hours or days later. This temporal dichotomy seems to be due to different phases of the overall consolidation process (Alberini, 2011). Studies showed that memory consolidation is not based on a unique, single process of molecular consolidation, and that once stabilized against these interferences, memories can again revert to a labile state for a limited period of time if retrieved or reactivated. These reactivated memories over time once again become stable and insensitive to disruption—a process that has been termed reconsolidation (Alberini, 2005; Alberini et al., 2006; Dudai, 2004; Nader, 2003; Sara, 2000). These findings on memory reconsolidation revolutionized the way we think about long-term memory formation, storage, recall, and stability, or actually the unstable, dynamic nature of memory traces (Alberini, 2010). Knowing that memories after retrieval are fragile, changeable, and disruptable is important for many reasons. For example, in addition to gaining a better understanding of mental processes, this knowledge provides an opportunity to develop more accurate therapeutic protocols in mental health, including psychoanalysis and psychotherapy, that specifically target the intrinsic features and mechanisms of mnemonic processes. Following the rediscovery of memory reconsolidation, a few studies went on to examine the effect of employing behavioral or the combination of behavioral and pharmacological methods for treating psychopathologies such as PTSD and addiction (Surís et al., 2013). These findings about reconsolidation trace reactivation are important for the dialogue between neuroscience and psychoanalysis, because the molecular mechanisms of neuronal plasticity, which allow the recording of experience in the form of synaptic traces within the nervous system, introduce a discontinuity between the trace and the initial perceptual experience. Regardless of any determinism, and insofar as the reassociation of traces leads to the production of something unique, the neurobiological fact that the structure of the nervous system changes depending on the impact experience has on neuronal connections paves the way for the emergence of singularity. It appears, therefore, that psychoanalysis and neuroscience can meet, around the shared observations that experience is inscribed and leaves a trace, but that, paradoxically, this inscription distances us from the initial experience.

However, we consider that to approach the field of trauma through the prism of a dialogue between psychoanalysis and neuroscience based solely on the implications of the recording of traces and their reassociations, runs the risk of interpreting post-traumatic syndromes as purely pathologies of the memory. So we propose to approach the field of trauma from the perspective of the interdisciplinary model ‘R – S’ (Representation – Somatic State). This model is based on two observations: that the inscription of traces—be they understood as memory traces in the psychoanalytic tradition, or as synaptic traces in the field of neuroscience – implies a discontinuity with respect to initial perceptual experiences; that the inscription itself cannot be dissociated from the perception of certain somatic states, the internal states of the body, that fall within the field of interoception (Ansermet and Magistretti, 2010; Tran The, 2022). Representations ‘R’, resulting from the exteroceptive perceptions, are linked with the representations resulting from the interoceptive perception of somatic states ‘S’ that have been the subject of concomitant and synchronic perception. The primordial nature of the organism’s interoceptive perception of states—for example, a sensation of pleasure or displeasure—that accompany external perceptions plays an important role in psychoanalytic theory (Freud, 1900; Freud, 1925). It is also the subject of a number of recent neuroscientific investigations (Damasio, 1994; Prochiantz, 2000; Craig, 2002, 2003). Thus, these two disciplines find common ground around a homeostatic understanding of psychic functioning—particularly that of thought. The representations brought to bear by our thoughts play a role in regulating our somatic states. Hence, the homeostatic function of the trace emerges as a point of convergence between psychoanalysis and neuroscience.

This hypothesis can also appear similar with Karl Friston’s Free Energy Principle (FEP). The FEP proposes that living organisms, as organized systems in a state of equilibrium with their environment, need to minimize their free energy, which is to say they need to resist the natural tendency to disorder (Friston, 2009). As has been suggested by Mark Solms, this minimizing of the free energy is similar to a homeostatic understanding of the regulation of the physiological states, which would be one of the most basic functions of consciousness-based somatic and affective states (Solms, 2013; Solms and Friston, 2018). In Solms’ words, “…the long-sought mechanism of consciousness is to be found in an extended form of homeostasis” (Solms, 2019).

## 8 The economic understanding of trauma reformulated as part of the ‘R – S’ model

Based on the R – S model, we can reformulate the psychoanalytic theory of trauma within an interdisciplinary framework: trauma should primarily be understood as assimilable to a surfeit in S that would initially defeat the homeostatic function of R. Indeed, the Freudian definition of trauma appears to belong primarily to an economic view of metapsychology. This economic perspective had its origins in the concept of ‘quantity’ of neuronal excitation found in *A Project for a Scientific Psychology* (Freud, 1895a,b). This definition acquired a fundamental importance in Freudian metapsychology once Freud set aside any reference to a physiology of the nervous system (Tran The et al., 2018). The concept of quantity was thereafter understood as a form of psychic excitation, or energy—also called *libido*. It was on this basis that, in 1916–1917, Freud proposed an economic definition of trauma, ‘the term “traumatic” has no other sense than an economic one. We apply it to an experience which within a short period of time presents the mind with an increase of stimulus too powerful to be dealt with or worked off in the normal way, and this must result in permanent disturbances of the manner in which the energy operates’ (Freud, 1916-1917a, p. 275). Returning to this economic model as part of a dialogue between psychoanalysis and neuroscience, makes it possible to shift the focus away from a view of trauma that reduces it to a phenomenon essentially involving an event and a memory—either trace, or memory left by that event.

When, at the end of the First World War, Freud and his colleagues found themselves confronted with the clinical problem of war neuroses, they postulated that in this pathology —involving an overflow of excitement—it would so happen that the binding capacities of psychic energy were deactivated. It should be pointed out that Freud explicitly distinguished these clinical pictures from those of transference neuroses such as hysteria, ‘The symptomatic picture presented by traumatic neurosis approaches that of hysteria in the wealth of similar motor symptoms, but surpasses it as rule in its strongly marked signs of subjective ailment (in which it resembles hypochondria or melancholia)’ (Freud, 1920, p. 12). Freud used the term ‘traumatic neurosis’—first introduced by Oppenheim—and this concept is often used in the psychoanalytic field. Yet it may seem a poor choice, and a confusing one, since the term ‘neurosis’ tends to swiftly equate traumatic neuroses with transference neurosis. When in fact Freud believed that the traumatic syndrome shared a libidinal structure with those pathologies termed ‘narcissistic’. Freud argued that in traumatic neurosis the mechanism was a process comparable to conditions that he had grouped under the term ‘narcissistic’ pathologies—a group in which he places both ‘severe neuroses’ such as melancholia, and psychoses such as dementia praecox and paranoia. The common link between these pathologies and traumatic neurosis was that the libido—or ‘sexual energy’—involved was not objectual but narcissistic and would flow back onto the ego, exceeding the capacities of the psychic apparatus to bind and control it (Freud, 1919, pp. 209–210).

At the 1918 Fifth International Psycho-Analytical Association Congress in Budapest, the Freudian hypothesis of an essentially narcissistic libidinal economy in traumatic neurosis was shared by all. Ferenczi described the traumatic process using, word for word, a formulation that Freud had used not about traumatic neuroses but to describe Schreber’s case,^7^ ‘in consequence of the shock, which has been experienced once or repeatedly, the interest and sexual hunger (libido) of the patient is withdrawn from the object into the ego. There thus comes about a damming-up of the sexual hunger (libido) in the ego, which is expressed in those abnormal hypochondriacal organic sensations and over-sensitiveness’ (Ferenczi, 1921, p. 18). Ferenczi added that a subject who is ‘already predisposed to narcissism will of course sooner fall a victim to a traumatic neurosis’ but that ‘no one is entirely immune from it, since the stage of narcissism forms a significant fixation point in the development of the sexual hunger (libido) of every human being’. The perspective of a libidinal process in traumatic neurosis *and* so-called ‘narcissistic’ pathologies such as psychosis does not, therefore, imply the idea of an etiological similarity. It is only a description in economic terms that highlights the same movement of excitement flowing back on the ego.

We might point out that these 1918 descriptions of the narcissistic libidinal process occurring in traumatic neurosis are comparable to the clinical pictures that Freud had grouped under the term ‘actual’ or ‘current’ neuroses—wherein neurasthenia and anxiety neurosis were grouped together, and to which Freud would later add hypochondria. These latter, contrary to hysteria or obsessive neurosis, belonged to a sexual etiology that was not infantile but *current* (Freud, 1898). This was an etiology wherein the anxiety had its origin in the accumulation of an excitation of somatic origin, not a psychic one. In current neurosis, excitement was not successfully elaborated psychically through representations. Rather, it remained distanced from the psychic, manifesting itself essentially through somatic symptoms. Unlike the symptoms of hysterical conversion though, these did not stem from a formation which was a compromise between opposing representations (Freud, 1895b).

When integrated within the R – S model, these parallels between post-traumatic syndromes and the process of libidinal withdrawal in psychosis, or the ‘somato-somatic’ short-circuiting found in current neurosis, enable us to highlight how, in the first instance, the traumatic process involves an excess S that can no longer be subjected to homeostatic regulation or the *binding* of free energy (Friston et al., 2006; Friston Karl, 2010). This ‘surfeit of the living’ can be likened to the body’s state of ‘absence of unity’ characteristic of very early infancy (Ansermet and Magistretti, 2010). Indeed, Freud had maintained ‘that a unity comparable to the ego cannot exist in the individual from the start’ (Freud, 1914, p. 77). This was an idea that Lacan (2007) further developed in his discussion of the ‘mirror stage’. In Lacan’s argument, the early stages of life are characterized by a bodily state of fragmentation and vital impotence. In this state the child is dominated by feelings of deep discomfort and distress, and cannot yet demonstrate unity in their interoceptive perceptions (Lacan, 2007). It is the advent of representation and language that will mediate the relationship to the raw reality of the body. Lacan later introduced the term ‘jouissance’ to emphasize that something of the initial ‘libidinal reserve’ nonetheless remains that cannot be encompassed by image and language. That is to say, it remains ‘profoundly invested at the level of one’s body, the level of primary narcissism’ (Lacan, 2014, p. 45).

In trauma, the phenomenon of redirecting the withdrawn narcissistic libido onto the ego can result in a symptomatology that first expresses itself in a purely somatic form. This is a process similar to the libidinal withdrawal that Freud had described in the early stages of Schreber’s disease, and in hypochondria (Freud, 1914). It can manifest as generalized anxiety, internal tensions, tremors, somatic pain without organic cause, or even paralysis as described by Oppenheim (2012) and Ferenczi (1921). These somatic symptoms may be a reversal to a primitive relationship with the fragmented body and its surfeit of *jouissance* that overwhelmed the subject.

As Dimitriadis (2021) pointed out, the subject’s first response in the case of confrontation with a brutal event “traumatically engages the body,” since, in psychological trauma, even when there is no bodily injury, the consequences directly involve somatic manifestations: palpitations, tightening in the stomach and throat, feeling of lack of air, sweating, etc. These first manifestations, comparable to a form of anti-homeostasis, could testify to somatic manifestations that have lost their connection with the metaphorical and metonymic processes of the unconscious (Dimitriadis, 2021). By reformulating the economic conception of trauma in the R-S model, it would thus initially be a loss of the capacity for association and reassociation of traces, the somatic states S manifesting themselves in a lack of binding with the R representations.

Another advantage of the economic interpretation of trauma formulated by Freud is its focus away from intrinsic nature of the event, to favor the relationship between the event and how it is understood by the subject. Counter to the hypothesis that the event would leave a traumatic trace on the subject we may say that the traumatic event is precisely that which *does not* form a trace. Hence, for Schreber, his appointment as president of the District Court of Dresden had a traumatic impact *because it was impossible to represent*. Lacan’s contributions are fundamental in shedding light on this argument. Lacan proposed rereading the Freudian concept of ‘trace’ in terms of ‘signifier’, given that the ‘phenomena that Freud is interested in are always language phenomena’ (Lacan, 1997, p. 156). In the Lacanian interpretation trauma is an experience over which the signifier has no hold, a form of ‘hole’ [trou] in the symbolic network. Hence the neologism ‘*troumatisme’* to describe that which cannot be symbolized. The original example of which—for both Lacan and Freud—was the emergence of the sexual—emergence that constitutes a ‘hole in the Real’ (Lacan, 1974). In that context, events as disparate in nature as the first encounter with sexuality, Schreber’s appointment, or the confrontation with mortality in a deadly situation, can appear similar when viewed from a structural perspective. Only the aftermath makes it possible to determine what *will have caused trauma* for a subject caught-up in their singularity.

## 9 The post-traumatic symptom: a subjective and creative process with a homeostatic function?

Although some of the somatic symptoms that a subject presents may give the appearance of passivity in the face of overwhelming life, or the delocalization *jouissance*^8^ in the body that occurs during trauma. In the context of the R – S model we can regard other symptoms as being active phenomena whereby the subject creates traces. These are phenomena where the production of R aims to ensure a binding function for the excess S. According to Freud, if in trauma the pleasure principle was initially disabled following the massive increase in excitation that overwhelmed the psychic apparatus, in a second phase the ego would take on the task of controlling the excitation by psychically binding the impinging quantities of excitation, to then diffuse them (Freud, 1920). Traumatic nightmares were an example of this type of symptom. They formed part of an attempt to create representations in order to bind the excess somatic states. Such dreams, which ‘lead them [the patient] back with such regularity to the situation in which the trauma occurred’, were not intended for the fulfillment of desire, rather their repetitive nature aimed ‘to master the stimulus retrospectively’ (Freud, 1920, p. 32). Lacan (1981) was to insist on this creative aspect of repetition, by stressing that in Freudian theory ‘repetition is not reproduction’ (p. 50), ‘repetition demands the new’ (p. 61).

Thus, as Hachet (2004) pointed out, following Freud, psychoanalysts have proposed a broader definition of the concept of “sublimation,” to show that creativity is not only at work in the diversion of sexual impulses towards socially valued activities. Creativity is also at work in the psychic elaboration of traumatic experiences. Sidney Stewart, a psychoanalyst and American veteran taken prisoner by the Japanese army in 1942, offered a first-person autobiographical account of the work of sublimation at work in the face of the inhuman, recounting not only his experience as the sole survivor of prison camps in Japan and Korea, where he experienced the atrocities of deprivation and humiliation, but also how he was able to use his creative abilities, in his work as an artist, to “survive the inhuman.” He offers an example of the possibilities of psychoanalytic treatment to stimulate and encourage such creative capacities (Stewart, 2009).

Overall, this interpretation can be applied to all the symptoms described by the DSM-III as ‘intrusions’ or as manifestations of traumatic flashbacks. These tend to become a pathognomonic sign of PTSD, or its neurophysiological basis. These phenomena, that are perceived as intrusions which repeat and are identical to the lived experience—whether they are diurnal flashbacks or repetition nightmares—can, through the prism of the R – S model, be reinterpreted not as linear *effects* of the cause that the event constitutes, but rather as subjective creations that introduce a discontinuity. Drawing on Freud, Ferenczi described these formations as ‘spontaneous attempts to cure on the part of the patient’ (Ferenczi, 1921, p. 20); a view similar to the Freudian hypothesis of delirium as an attempt at healing (Freud, 1911). Post-traumatic symptoms and delusional ideas would thus be attempts on the part of the subject to process the displacement of *jouissance* they experience. Repetition compulsion would in part serve this process of binding the trauma’s surfeit of excitation S, and traumatic nightmares were part of this process. This model has been taken up in contemporary work within Friston’s model—where dreams have been equated with a process of minimizing free energy and reducing complexity (Hopkins, 2016).

However, Freud had highlighted that the repetition compulsion cannot be conceived only as serving the process of binding—and by extension the pleasure principle that aims to ‘keep the quantity of excitation present in it as low as possible or at least to keep it constant’ (Freud, 1920, p. 9). In addition to the aspect of controlling excitation this compulsion also contained a more archaic component, linking it to the workings of the death drive. Flashbacks as a symptom cannot therefore be entirely situated within an attempt at healing. Sometimes repetition is a dead end, and constitutes what Guy Briole described as a ‘repeated failure of the attempt to shift toward meaning […] leaving the subject with a driving *jouissance* that is repeated through its terrifying threat’ (Briole et al., 1994) [our translation]. The processing of the real by the symbolic then becomes inoperative.

How can lines of research emerge from this aporia, shared by psychoanalysis and neuroscience, concerning what constitute the nucleus of trauma? The example of the French writer Louis-Ferdinand Céline is particularly enlightening for this purpose (Loisel and Saguin, 2021). The *maréchal des logis*^9^ Destouches of the 12th Cavalry Regiment—who became known under his pen name of Céline—was just 20 years old when the First World War broke out. Promised to a peaceful career as a tradesman, Destouches found himself confronted with the brutality of a war, the tactical absurdities of which appear very clearly in his letters to his parents (Céline, 2009). Like many *Poilus* [WWI French infantry men], writing was a necessity that ensured a lifeline to those back home. It also provided a time for perspective and reflection, a fragile grip on the extreme, and a link with the self. Destouches’ correspondence (Céline, 2009) gives us original material for understanding the evolution of the young soldier thoughts. In addition to the attrition brought on by apparently aimless marches and countermarches, we see the experience of powerlessness in the face of horror and carnage. On the battlefields of Flanders, on 25 October 1914, Destouches was wounded in the arm by a German bullet. Anomie and stupor are what stand out. Destouches described failing words and passivity in the face of pain, as if he were outside his own body. Then, a few days later, on 5 November 1914, a letter written from his bedside to his father, attested to a state of acute stress dominated by hypervigilance and flashbacks (Céline, 2009, p. 120). More than the bodily intrusion, the pain, the fear of infection and possible amputation, it was the psychological impact of the war that seems to have radically influenced this destiny. In the first instance it was a case of shielding himself from a possible return to the battlefield, by leaving for an administrative assignment in London in 1915; but very quickly it became primarily a need to flee the conformity of his previous world and to stand by his values. His time in London was followed by a decision to take up a position as supervisor in a rubber plantation in Cameroon. In this exotic territory, Destouches engaged in trading. More importantly though, he read: medical books, philosophy, literature, everything he could lay his hands on. He felt the need to have a grasp of himself, to re-discover himself, to be re-born. The trauma from which he suffered, the flashbacks, these had reshuffled the cards of what until then had seemed to constitute his self. Medical studies, but especially writing, appeared to be a solution.

Alongside his medical studies, he pursued a rich correspondence. Then, in 1932, *Voyage au bout de la nuit* [Journey to the End of the Night] was published to great acclaim. The first part of the novel presents the experience of combat as life defining, as a driving force. The incipit ‘It all began just like that’ hovers over the battlefield and his wounding (Céline, 1966, p. 7). This literary processing of trauma operates through an active process of transformation of the meaning of experience, a rewriting. This rewriting included his own childhood, which he reinterpreted in *Mort à crédit* (1936) [Death on the Installment Plan] as inevitably leading to the shock of 1914 (Céline, 1938). The whole of Céline’s *oeuvre* is characterized by the deconstruction of language, seen as insufficient in its classical state to resonate with a traumatized life experience. Trauma is lodged in Céline’s style, or in the typography. It breaks the syntax and vocabulary, with the recurrence of ellipses echoing the lingering rhythm of the unspeakable.

Céline’s story would be creditable if we did not have to evoke the anti-Semitic pamphlets that he published in 1937. His sense of identity shaken by a failure to win the Goncourt Prize in 1932, then by the mixed reception that *Mort à Crédit* received in 1936, it appears that Céline became terrified by the prospect of a return of war in Europe. The subjective solution he had found was being threatened from all sides. Fiction, even if it made it possible to manipulate the author’s complexes, could do nothing against what was looming in external reality, and it also struggled to counter what was reawakened in his internal world. The figure of the anti-Semite constituted an opportunistic and ready-made identification; one that had the advantage of relocating hatred and destructiveness on an object designated as responsible for the war. In the manner of an attempt to relocate *jouissance* in the Other, in a paranoid delusion, Céline, intoxicated by this surge of violence, spewed out his hatred, forever disfiguring his *oeuvre* with this sordid ejection (Saguin and Loisel, 2019). It may be argued that designating a persecuting figure in this way can give form and meaning to symptoms described by the DSM-III as akin to a sense of threat, hypervigilance, or a withdrawn attitude. These would be attempts to guard against the external world, where the surfeit of *jouissance* is located.

The example of Céline is particularly enlightening, since it reflects the subjective journey that takes place after a traumatic encounter. In the R – S model, we have proposed that in the case of trauma, somatic excitation fails to bind using the registers of representation and language. Céline, whose experience of war led him to a major existential breakdown, leaving him plagued by lasting and recurrent flashbacks, came up with a subjective solution through his writing. This was a solution that arose as an attempt to cope with the surfeit of excitation. But this *pharmakon* proved to be twofold, both a remedy siding with the life drive, and a deadly poison. This is evidenced by the episode of the pamphlets, where the position of *jouissance* necessitated the invention of a figure of the Other interpreted as a persecutor.

We should be emphasized that by finding a subjective solution to the excess of S in trauma—be it overflow of excitation or delocalization of *jouissance*—the diagnosis of PTSD can appear, today, to be a collective solution: insofar as it gives a name to the unspeakable reality of trauma and, for the subject, enables a new inscription in the social bond. The PTSD diagnosis then plays a role similar to that described by Freud in *The Future of an Illusion*, where a collective fiction, such as religion, makes it possible to treat the absence of meaning inherent to the human condition. It does so by offering the subject a set of off-the-peg representations (R) to process the surfeit of S. But as Freud points out, this ‘acceptance of the universal neurosis spares them the task of constructing a personal one’ (Freud, 1927, p. 44). In some cases, this solution can also arise because of the espousal of a new identity, made possible by the social recognition of the status of victim that the diagnosis of PTSD implies. Assoun described this subjective position of ‘injured party’ [préjudicé], as instrumental in structuring the subject’s speech and action; and more generally their ‘type of unconscious life, as well as their way of experiencing the world, and their relations with others’ (Assoun, 2012) [our translation]. By positioning themself as the object of the Other’s injurious action, the subject manages to locate the *jouissance* outside themself. This is a possible means for managing the overflow of trauma; and the authentication of this position by a discourse, whether social or medical, makes it possible to maintain the inscription of the subject within a social bond.

## 10 Conclusion

The Freudian interpretation of trauma centers on an economic approach and foregrounds the concept of deferred action. It accounts for both the subject’s structure and history, as well as their creative potentialities to respond to trauma. Thus, it introduces a discontinuity between event and symptom. The diagnosis of PTSD introduced in 1980 by the DSM-III, however, while it certainly contributed to a paradigm reversal in regard to the social recognition of victims, tends to approach trauma from a deterministic, linear, and continuist perspective. In this, it is antithetical to the psychoanalytic model.

Despite their differences, we have outlined a possible dialogue between psychoanalysis and contemporary neuroscientific research, based on our own R – S model. This dialogue involves a return to an economic understanding of trauma, and studies the libidinal dynamics at work in its process. Based on our model, trauma can be understood as an overflow of somatic states (S), the surfeit of which would not be regulated by the usual homeostatic function of representation (R) and language. Using Lacanian theory, and what we have characterized as a delocalization of *jouissance* that is primarily expressed through the body, some post-traumatic symptoms can be understood as the subject’s attempt to accommodate that surfeit of S. Thus, symptoms, instead of being linked to an effect produced directly by the traumatic event, would be in discontinuity with that event. Consequently, symptoms should be understood as the fruit of the subject’s unique creativity, their singular ‘attempt at healing’. The example of the function fulfilled by writing in the life of Céline, can be likened to such an attempt to accommodate *jouissance*.

The approach we propose is of interest for the therapeutic management of trauma as it encourages a shift away from a vision of post-traumatic symptoms as dysfunctions, as pathologies of memory, or as a deficit of emotional regulation. The dialogue between psychoanalysis and neuroscience around an economic conception of trauma based on the R – S model invites clinicians to focus on the possibilities presented by the subject’s unique response. It looks beyond determining factors. This approach reinstates the fundamental primacy of listening to the subjective solutions invented by the subject. Despite their pathological aspect, symptoms must be considered in their function for the subject’s economy. It is then that their creative dimension will emerge. In the words of Canguilhem, the pathology is ‘an alteration such that it constitutes a new way of life for the organism, new behavior which prudent therapy must take into account’ (Canguilhem, 1966, p. 84).

## Data Availability

The original contributions presented in the study are included in the article/supplementary material, further inquiries can be directed to the corresponding author.

## Footnotes

^1^We have taken the liberty to change “instinctual” for “driving” referring to that change in Solms’s Revised Standard Edition.

^2^Reference to the ‘middle term’ of a syllogism, enabling the passage from the premises to the conclusion (St. Thomas Aquinas).

^3^An intelligence which could comprehend all the forces by which nature is animated and the respective situation of the beings who compose it—an intelligence sufficiently vast to submit these data to analysis—it would embrace in the same formula the movements of the greatest bodies of the universe and those of the lightest atom; for it, nothing would be uncertain and the future, as the past, would be present to its eyes’ (Laplace, 1902, p. 4).

^4^Endowment and chance.

^5^The Freudian *Nachträglichkeit*, ‘deferred action’ in English translations. For the Freudian concept of *Nachträglichkeit*, Lacan introduced the French term ‘après-coup’. The Standard Edition of the works of Freud uses ‘deferred action’ for *Nachträglichkeit*, both terms have been used in this article.

^6^We have taken the liberty to change ‘instinct’ for ‘drive’ referring to that change in Solms’s Revised Standard Edition.

^7^‘The Patient has withdrawn from the people in his environment and from the external world generally the libidinal cathexis which he has hitherto directed onto them. Thus everything has become indifferent and irrelevant to him […]. The end of the world is the projection of this internal catastrophe; his subjective world has come to an end since his withdrawal of his love from it’ (Freud, 1911, p. 70).

^8^“Delocalization *jouissance*” is a Lacanian concept referring to Freud’s hypothesis that psychosis stemmed from a libidinal process in two phases. The initial process of psychosis consisted in massive withdrawal of the libido (or quantity of psychic energy) into the ego and the body itself. This was the first phase of the psychotic process, dominated by significant alteration in the perception of the body (Freud, 1914).

^9^This rank in the French Army may be translated by “sergeant.”
